# Impact of a health education program on knowledge and quality of life among patients undergoing cholecystectomy in public teaching hospitals in Erbil

**DOI:** 10.3389/fsurg.2026.1825682

**Published:** 2026-05-12

**Authors:** Burhan Izzadin Sabir, Yousif Mohammed Younis

**Affiliations:** Adult Nursing Department, College of Nursing, Hawler Medical University, Erbil, Iraq

**Keywords:** cholecystectomy, gallstone patients, gastrointestinal quality of life (GIQLI), health education program, Iraq, patients knowledge

## Abstract

**Background and aim:**

Gallstone disease (GSD) is one of the most common causes of biliary tract disorders and represents a major surgical health problem worldwide. Gallstones affect approximately 10%–15% of the adult population in developed countries and are associated with considerable morbidity and healthcare burden. Despite the high frequency of cholecystectomy procedures, many patients lack adequate knowledge regarding the disease, postoperative care, and lifestyle modifications, which may negatively affect recovery and quality of life. This study aimed to assess the impact of a health education program on knowledge and quality of life among patients undergoing cholecystectomy in surgical wards at public teaching hospitals in Erbil City, Iraq.

**Method:**

This quasi-experimental study was conducted from September 1st, 2024, to February 13th, 2025, across two major public teaching hospitals in Erbil City, Iraq, using a convenience sampling technique. Participants were allocated into an intervention group (*n* = 100) and a control group (*n* = 100) based on the hospital site to minimize cross-contamination of the intervention. Baseline sociodemographic and clinical characteristics, as well as standard surgical and discharge protocols, were systematically compared between the two hospital sites to assess comparability. Data were collected using three structured instruments: the patients' medical data form, a researcher-developed knowledge questionnaire constructed based on current clinical practice guidelines for cholecystectomy and validated through expert content review (CVI = 0.92) by a panel of five surgical and nursing experts, and the Gastrointestinal Quality of Life Index (GIQLI), along with a sociodemographic form. Statistical analyses were performed using SPSS version 26.0. Descriptive statistics, Chi-square tests, independent t-tests, paired t-tests, and logistic regression analysis were used to assess group differences and predictors of knowledge improvement.

**Results:**

A total of 200 patients participated in the study. The findings showed significant improvements in the intervention group compared with the control group. Post-intervention knowledge scores increased significantly in the intervention group (28.09 ± 2.51) compared with the control group (14.15 ± 2.79) (*p* < .01). Similarly, the total gastrointestinal quality-of-life score improved markedly in the intervention group (119.15 ± 10.02) compared with the control group (79.05 ± 14.05) (*p* < .01). Logistic regression analysis indicated that participation in the educational program was the strongest predictor of knowledge improvement (AOR = 187.34, *p* < .01).

**Conclusions:**

The health education program significantly improved patients' knowledge and gastrointestinal quality of life following cholecystectomy. Implementing structured patient education programs in surgical wards may enhance postoperative recovery, promote self-care behaviors, and improve overall patient outcomes in public hospitals.

**Clinical Trial Registration**: clinicaltrials.gov, identifier NCT07380776.

## 1 Introduction

Gallbladder disease is a common yet often underestimated global health concern, the incidence and complications of which have long been overlooked in surgical and community health research (1). Globally, gallbladder and biliary tract diseases account for nearly 20 million new cases annually, with about 10%–15% of the adult population in developed nations and up to 25% in developing regions affected by gallstones (1, 2). Inadequate health education and the absence of structured preoperative counseling programs have contributed to inconsistent knowledge among patients and variable recovery outcomes following cholecystectomy procedures (3, 4). Although gallbladder pathology occurs worldwide, it predominantly affects middle-aged adults and populations adopting high-fat, low-fiber dietary habits that predispose to gallstone formation (5). In Iraq, hospital-based records show that cholecystectomy represents nearly 12%–18% of all elective abdominal surgeries, with a notable increase among females aged 30–55 years (6). In the Kurdistan Region specifically, perioperative patient education remains limited, and surgical wards in public teaching hospitals often lack standardized protocols for health education, contributing to suboptimal patient preparedness and variable recovery outcomes. A global strategy to standardize perioperative patient education and enhance surgical recovery outcomes was first encouraged by WHO in 2002; however, gallbladder disease–specific health education initiatives were not included in this framework (7). Comprehensive patient-centered education protocols for cholecystectomy were only proposed nearly two decades later, through the WHO 2021–2030 people-centered health care agenda (8). Consequently, patient education before gallbladder surgery remains one of the most neglected components of perioperative nursing care in many developing settings.

Unlike most surgical procedures that involve transient physiological effects, cholecystectomy permanently alters bile flow and digestion, leading to variable gastrointestinal adaptation among patients (9, 10). After surgery, nearly 40% of patients experience mild to moderate dyspeptic symptoms, while up to 15% report long-term post-cholecystectomy syndrome characterized by bloating, diarrhea, and intolerance to fatty foods (11). This distinction emphasizes the need for targeted health education to improve postoperative adaptation and symptom management. Most patients undergoing cholecystectomy experience temporary abdominal discomfort, flatulence, or dietary intolerance, which, without proper guidance, can reduce overall satisfaction and quality of life (11, 12). By contrast, patients with adequate preoperative counseling are more likely to adopt appropriate postoperative dietary habits and recover smoothly, demonstrating better outcomes in knowledge retention, self-care behavior, and comfort (4). Recent studies across Asia and the Middle East have reported that structured preoperative education can improve postoperative knowledge scores by 35%–50%, and reduce hospital stay duration by up to 1.5 days (13). Vulnerable patients with chronic diseases, limited literacy, or anxiety are particularly at risk of poor postoperative adaptation, delayed wound healing, or decreased confidence in recovery (10). These factors collectively contribute to significant disparities in recovery experiences and highlight the urgent need for structured educational programs within surgical wards (14). Furthermore, evidence indicates that when patients receive comprehensive nursing education, readmission rates drop by nearly 20%, and self-reported confidence in managing daily activities increases substantially.

Current perioperative care primarily focuses on medical and technical aspects of surgery, while patient education and psychosocial support often receive less attention (14). In many public hospitals, preoperative sessions last fewer than 10 min and are limited to procedural explanations rather than lifestyle guidance (15). Standard postoperative instructions may vary widely between hospitals, and their effectiveness is rarely evaluated systematically (16). In the absence of evidence-based educational programs, patients depend mainly on informal advice or inconsistent nurse-led guidance during short hospital stays (17). This inconsistency leads to wide variations in postoperative recovery, with some patients achieving full functional independence within one week, while others experience persistent discomfort or dietary restriction for several months (18). Moreover, unstandardized approaches to patient teaching can hinder adherence to postoperative care instructions and dietary modifications (7). Several interventional studies have shown that structured education programs can improve patient knowledge, reduce anxiety, and enhance satisfaction with surgical outcomes (10). However, limited data exist in Iraq and similar resource-constrained settings, where patient education is rarely incorporated into the standard cholecystectomy care pathway.

Given the lack of context-specific educational strategies and the growing burden of gallbladder surgeries in the region, there is a critical need for effective interventions that promote informed participation in health care. Health education programs led by trained nursing professionals have demonstrated significant benefits in improving knowledge and enhancing recovery-related quality of life in various surgical populations (19). Preliminary evidence suggests that such interventions can foster better self-management, faster rehabilitation, and lower postoperative complications among abdominal surgery patients (4). These findings have encouraged further exploration of nurse-led programs targeting patients undergoing cholecystectomy, with emphasis on improving both cognitive and functional outcomes. The present study contributes a novel perspective by combining a culturally adapted, dual-language (Kurdish and Arabic) education protocol with the assessment of specific predictors of knowledge improvement in an under-studied Iraqi public teaching hospital setting, addressing a clear gap in the regional perioperative literature. Therefore, the present study aimed to evaluate the impact of a structured health education program in improving patient knowledge and overall quality of life following cholecystectomy in public teaching hospitals in Erbil.

## 2 Research question

Does a structured health education program improve patient knowledge and gastrointestinal quality of life among individuals undergoing cholecystectomy in public teaching hospitals in Erbil?

## 3 Methods

### 3.1 Study design, setting, period, and sampling

This quasi-experimental study was conducted using a convenience sampling technique in two major public teaching hospitals in Erbil City, Iraq: Hawler Teaching Hospital and Rizgary Teaching Hospital. Convenience sampling was deemed appropriate due to the limited recruitment window, the operational constraints of both hospitals, and the need to enroll consecutive eligible patients scheduled for cholecystectomy. To minimize cross-contamination of the intervention, the intervention group was recruited from one hospital and the control group from the other. The two hospitals were selected because they share comparable characteristics: both are public teaching hospitals affiliated with Hawler Medical University, follow similar standard surgical protocols and discharge criteria for laparoscopic cholecystectomy, are staffed by surgeons and nurses with comparable training, and serve patient populations with similar sociodemographic profiles.

### 3.2 Sample size

The sample size was calculated using G*Power software (version 3.1.9.4) based on the *t*-test for the difference between two independent means (two groups). The calculation assumed an effect size (d) of 0.5, an alpha error probability of 0.05, and a power (1–β) of 0.95. Based on these assumptions, the required sample size was 200 participants, with 100 patients allocated to the intervention group and 100 to the control group.

### 3.3 Inclusion/exclusion

The inclusion criteria for participants were adult patients diagnosed with gallstone disease confirmed by ultrasound and clinical examination who were scheduled to undergo cholecystectomy. Eligible participants were required to be functionally independent, free from any diagnosed mental or psychiatric disorders, and willing to participate in the study. In addition, participants had to be capable of understanding and attending the educational sessions, which were delivered in Kurdish and Arabic.‏ The exclusion criteria included patients with psychiatric disorders, those who were unwilling to participate, or individuals who were unable to complete the educational program due to cognitive or physical limitations. Patients who missed consecutive educational intervention sessions for any reason were also excluded. Furthermore, individuals with professional health-related qualifications were excluded from the study to avoid potential bias related to prior medical knowledge.

### 3.4 Study tools and data collection

Data were collected using a structured questionnaire consisting of four main parts. The first part collected sociodemographic characteristics of the participants, including age, gender, educational level, marital status, place of residence, occupation, family income, and whether the participants had previously received information about gallbladder disease and the source of that information. For female participants, the number of pregnancies was also recorded.‏

The second part assessed the medical characteristics of the participants. This section included variables related to gallbladder disease such as the duration of cholelithiasis, method of diagnosis, whether the diagnosis was incidental (asymptomatic) or symptomatic, previous hospital admissions due to gallbladder problems, number of hospital admissions, family history of gallstones, relationship with family members, body mass index (BMI), family history of obesity, presence of comorbidities (such as hypertension, diabetes mellitus, kidney disease, hyperlipidemia, and hemolytic anemia), smoking status and type of smoking, alcohol consumption, current symptoms, duration of symptoms, characteristics of abdominal pain, and surgical indications. These data were obtained from the patients' medical records.‏

The third part assessed the patients' knowledge regarding gallstones and cholecystectomy. This section consisted of 36 items distributed across five domains: general information about the gallbladder (8 items), information about cholecystectomy surgery (5 items), preoperative preparation (9 items), postoperative care (8 items), and discharge instructions (6 items). The instrument was developed by the researchers based on current evidence-based clinical practice guidelines for gallstone disease and cholecystectomy, including the Tokyo Guidelines and recent peer-reviewed perioperative nursing literature. To establish content validity, the questionnaire was reviewed by a panel of five experts comprising two general surgeons, two academic nursing faculty members specializing in adult surgical nursing, and one medical educator. Each item was rated for relevance, clarity, and necessity. The Content Validity Index (CVI) at the item level (I-CVI) was calculated, with all items achieving an I-CVI ≥ 0.80 and an overall scale-level CVI (S-CVI) of 0.92, indicating excellent content validity. Items were revised based on expert feedback before pilot testing.‏

The fourth part evaluated patients' quality of life using the Gastrointestinal Quality of Life Index (GIQLI). The GIQLI is a standardized instrument consisting of 36 items designed to measure quality of life before and after gastrointestinal surgery. It includes five domains: gastrointestinal core symptoms (19 items), emotional function (5 items), physical function (7 items), social function (4 items), and one item assessing the patient's subjective evaluation of treatment. Each item is scored on a five-point Likert scale ranging from 0 (least desirable condition) to 4 (most desirable condition). The total score ranges from 0 to 144, with higher scores indicating better quality of life. For interpretation, gastrointestinal core symptoms scores range from 0 to 76, emotional function from 0 to 20, physical function from 0 to 28, social function from 0 to 16, and treatment response from 0 to 4. The questionnaire was originally developed in English and translated into Kurdish and Arabic using the forward–backward translation method to ensure linguistic accuracy and cultural appropriateness.‏

Data were collected through face-to-face interviews with participants who met the inclusion criteria. Each participant required approximately 15–20 min to complete the questionnaire. The educational intervention was delivered through individual instruction by the researcher in the surgical departments of both teaching hospitals. Each educational session lasted approximately 40–45 min and was conducted before and after the cholecystectomy procedure. The intervention used a combination of pedagogical methods, including verbal explanation supported by visual aids (illustrated slides and anatomical diagrams), interactive question-and-answer dialogue, demonstration of wound care and breathing exercises, and teach-back techniques to verify comprehension. Each participant in the intervention group also received a structured printed educational booklet (in Kurdish or Arabic, according to preference) covering gallstone disease, the cholecystectomy procedure, preoperative preparation, postoperative self-care, dietary guidance, warning signs, and discharge instructions. The booklet served as a reinforcement resource during hospitalization and after discharge. Post-intervention assessments were conducted at 4 weeks following the cholecystectomy procedure to allow sufficient time for initial recovery and knowledge consolidation.

### 3.5 Pilot study

The study questionnaire was initially tested with a group of 20 patients between June 1st, 2024, and July 1st, 2024. The pilot testing aimed to assess the internal consistency and reliability of the questionnaire items before their application in the main study. The pilot study was conducted using Cronbach's alpha to evaluate reliability (20). The patient's knowledge demonstrated a Cronbach's alpha of 0.85 (very good), the Gastrointestinal quality of life index (GIQLI) showed a Cronbach's alpha of 0.94 (excellent), It is important to note that the data from the pilot study were excluded from the final analysis.

### 3.6 Measures

#### 3.6.1 Sociodemographic characteristics

The first section of the questionnaire collected sociodemographic information of the participants, including age, gender, educational level, marital status, place of residence (urban, rural, or suburban), occupation, whether the participant had previously received information about gallbladder disease, the source of that information, family income, and the number of pregnancies for female participants.

#### 3.6.2 Medical data

The second section of the questionnaire included medical data related to patients with gallstone disease undergoing cholecystectomy. This section collected information on the duration of cholelithiasis, method of diagnosis, whether the condition was diagnosed incidentally (asymptomatic) or presented with symptoms, previous hospital admissions due to gallbladder problems, and the number of hospital admissions related to gallbladder disease. It also included family history of gallstones, patient relationships with family members, body mass index (BMI), family history of obesity, and the presence of comorbidities such as hypertension, diabetes mellitus, kidney disease, hyperlipidemia, hemolytic anemia, and other medical conditions. Additional information was collected regarding smoking status and type of smoking, alcohol consumption, current symptoms experienced by the patient, duration since symptom onset, characteristics of abdominal pain, and the indication for surgery. These clinical data were obtained from the patients' medical records.

#### 3.6.3 Quality of life health status

The fourth tool used in this study was the Gastrointestinal Quality of Life Index (GIQLI), originally developed by Eypasch et al. in (1994). The instrument consists of 36 items designed to assess the quality of life of patients with gastrointestinal conditions. It includes four domains: gastrointestinal core symptoms (19 items), emotional function (5 items), physical function (7 items), and social function (4 items), in addition to one item evaluating the patient's subjective assessment of treatment effectiveness. Each item is rated on a five-point Likert scale ranging from 0 to 4, where 0 represents the least desirable condition and 4 represents the most desirable condition, reflecting the patient's experiences during the preceding two weeks. The total score ranges from 0 to 144, with higher scores indicating better quality of life. For interpretation, the total gastrointestinal quality-of-life scores were categorized as follows: scores below 80 indicate very poor quality of life with severe burden, scores of 80–99 indicate poor to moderate quality of life, scores of 100–119 indicate good quality of life with good functional status, and scores of 120–144 indicate excellent quality of life. The “response to treatment” domain consists of a single item that asks patients to rate their overall satisfaction with the treatment received, scored from 0 (very unsatisfied) to 4 (very satisfied). The reliability of the GIQLI scale was assessed using Cronbach's alpha, which yielded a coefficient of 0.94, indicating excellent internal consistency.

### 3.7 Ethical approval and inform consent

This study was conducted in accordance with the Institutional Research Ethics Board requirements and the principles of the Declaration of Helsinki. Ethical approval was obtained from the Ethics Committee of the College of Nursing at Hawler Medical University. In addition, official permission was granted by the surgical departments of the participating teaching hospitals in Erbil City to conduct the study (Approval No. 2439, issued on August 28, 2024). Written informed consent was obtained from all participants prior to their participation in the study and before completing the questionnaires.

### 3.8 Statistical analysis

Data were analyzed using Statistical Package for the Social Sciences (SPSS) version 26.0 (IBM Corp., Armonk, NY, USA). Descriptive statistics were used to summarize the data, with frequencies and percentages reported for categorical variables (e.g., sociodemographic and clinical characteristics), and means with standard deviations presented for continuous variables. Chi-square tests or Fisher's exact tests were applied to examine associations between categorical variables, while independent samples *t*-tests were used to compare mean differences between the intervention and control groups. Paired *t*-tests were employed to assess within-group changes in knowledge and gastrointestinal quality-of-life scores before and after the intervention. The magnitude of the intervention effect was evaluated using Cohen's d effect size. In addition, binary logistic regression analysis was conducted to identify predictors of post-intervention knowledge improvement, with adjusted odds ratios (AOR) and 95% confidence intervals (CI) reported for the associated factors. Model adequacy was assessed using the Nagelkerke *R*^2^ statistic and the Hosmer–Lemeshow goodness-of-fit test. A two-tailed *p*-value of <0.05 was considered statistically significant for all analyses. To minimize confounding, baseline characteristics that differed between groups were included as covariates in adjusted logistic regression models. Missing data were minimal (<2% across all variables); cases with missing values were excluded from the respective analyses on a pairwise basis. Missing data were minimal (<2% across all variables); cases with missing values were excluded from the respective analyses on a pairwise basis.

## 4 Results

### 4.1 Socio-demographic characteristics of participants

A total of 200 patients participated in this study, equally divided between the intervention group (*n* = 100) and the control group (*n* = 100). The mean age of the intervention group was 40.88 ± 11.88 years, while the control group averaged 44.31 ± 14.06 years, indicating a comparable age distribution (*p* = 0.29). Most participants were mid-life adults (40–64 years), representing 49% of the intervention and 47% of the control group. Females predominated in both groups, comprising 65% of the intervention and 75% of the control group (*p* = 0.12). The majority were married (83% vs. 85%), and most lived in urban areas (63% vs. 54%), both without statistical significance. Occupationally, housewives formed the largest subgroup (39% vs. 45%), followed by self-employed individuals (21% vs. 11%). Only 22% of the intervention group and 31% of the control group reported prior information about gallbladder disease, mainly obtained via the internet (12% vs. 16%). Most participants reported an insufficient family income (55% vs. 50%), and among female respondents, nearly half in the intervention group (49%) and one-third in the control group (32%) had no history of pregnancy. Detailed demographics and other variables are presented in Table 1.

**Table 1:** Socio-demographic data of intervention and control groups.

Variable	Classification	Intervention *n* = 100	Control *n* = 100	*p*-value
Age group based on life stages	Young Adult (18–39)	46 (46.00)	42 (42.00)	0.29 NS
	Mid-Life Adult (40–64)	49 (49.00)	47 (47.00)	
	Elderly (≥65)	5 (5.00)	11 (11.00)	
	Mean ± SD	40.88 ± 11.88	44.31 ± 14.06	
Gender	Male	35 (35.00)	22 (22.00)	0.12 NS
	Female	65 (65.00)	75 (75.00)	
Educational level	Illiterate	12 (12.00)	21 (21.00)	0.26 NS
	Basic school graduate	26 (26.00)	21 (21.00)	
	Secondary school graduate	23 (23.00)	15 (15.00)	
	Institution graduate	13 (13.00)	17 (17.00)	
	University graduate and more	15 (15.00)	11 (11.00)	
Marital status	Single	17 (17.00)	15 (15.00)	0.70 NS
	Married	83 (83.00)	85 (85.00)	
Residential area	Urban	63 (63.00)	54 (54.00)	0.74 NS
	Suburban	35 (35.00)	37 (37.00)	
	Rural	2 (2.00)	9 (9.00)	
Occupation	Governmental employee	16 (16.00)	22 (22.00)	0.70 NS
	Self-employed	21 (21.00)	11 (11.00)	
	Unemployed	4 (4.00)	3 (3.00)	
	Housewife	39 (39.00)	45 (45.00)	
	Retired	4 (4.00)	10 (10.00)	
	Military	9 (9.00)	2 (2.00)	
	Student	7 (7.00)	7 (7.00)	
Receiving information about gallbladder disease	Yes	22 (22.00)	31 (31.00)	0.15 NS
	No	78 (78.00)	69 (69.00)	
If yes, source of information	No	78 (78.00)	69 (69.00)	0.27 NS
	TV	0 (0.00)	2 (2.00)	
	Family/Friends	8 (8.00)	7 (7.00)	
	Healthcare staff	2 (2.00)	6 (6.00)	
	Internet	12 (12.00)	16 (16.00)	
Family income	Insufficient	55 (55.00)	50 (50.00)	0.50 NS
	Sufficient	45 (45.00)	49 (49.00)	
	Exceeds needs	0 (0.00)	1 (1.00)	
For female patients: number of pregnancies	No history of pregnancy	49 (49.00)	32 (32.00)	0.24 NS
	1–4 pregnancies	29 (29.00)	28 (28.00)	
	5–8 pregnancies	19 (19.00)	31 (31.00)	
	> 8 pregnancies	3 (3.00)	9 (9.00)	

NS, non-significant. Values are presented as frequency (percentage). *p*-values obtained using Chi-square or independent t-test where applicable. MS, Mean score; SD, Standard deviation. Statistical significance set at *p* < .05.

### 4.2 Medical characteristics of patients

The results showed that most participants had chronic cholelithiasis (55% in the intervention and 61% in the control group), and nearly all were diagnosed by ultrasound (99% vs. 100%). The majority were symptomatic (82% vs. 77%) and had a history of hospital admission (62% vs. 63%). Obesity was predominant (58% vs. 68%), and a family history of gallstones was frequent (64% vs. 57%). Significant differences were noted in family history of obesity (55% vs. 73%, *p* = 0.01), alcohol consumption (0% vs. 5%, *p* = 0.02), and symptom duration (<6 months: 60% vs. 48%, *p* = 0.03). Other medical factors and comorbidities showed no significant variation between groups (*p* > 0.05). For further details, see Table 2.

**Table 2:** Medical data of patients in the intervention and control groups.

Medical Data	Classification	Intervention *n* = 100	Control *n* = 100	*p*-value
Duration of diagnosis of cholelithiasis	<1 month (Acute)	15 (15.00)	14 (14.00)	0.67 NS
	1–3 months (Sub-acute)	30 (30.00)	25 (25.00)	
	>3 months (Chronic)	55 (55.00)	61 (61.00)	
Diagnosis method	Ultrasound	99 (99.00)	100 (100.00)	0.32 NS
	CT scan	1 (1.00)	0 (0.00)	
Diagnosed as	Asymptomatic	18 (18.00)	23 (23.00)	0.88 NS
	Symptomatic	82 (82.00)	77 (77.00)	
Previous hospital admission due to gallbladder problems	Yes	62 (62.00)	63 (63.00)	0.88 NS
	No	38 (38.00)	37 (37.00)	
Times admitted to hospital	None	37 (37.00)	36 (36.00)	0.32 NS
	One time	24 (24.00)	34 (34.00)	
	2–3 times	28 (28.00)	24 (24.00)	
	>3 times	11 (11.00)	6 (6.00)	
Family history of gallstones	Yes	64 (64.00)	57 (57.00)	0.31 NS
	No	36 (36.00)	43 (43.00)	
Relationship to affected person	No	36 (36.00)	43 (43.00)	0.19 NS
	Father	12 (12.00)	1 (1.00)	
	Mother	33 (33.00)	34 (34.00)	
	Brother	4 (4.00)	3 (3.00)	
	Sister	15 (15.00)	19 (19.00)	
BMI category	Underweight	1 (1.00)	0 (0.00)	0.34 NS
	Normal	11 (11.00)	8 (8.00)	
	Overweight	30 (30.00)	24 (24.00)	
	Obese	58 (58.00)	68 (68.00)	
Family history of obesity	Yes	55 (55.00)	73 (73.00)	0.01 S
	No	45 (45.00)	27 (27.00)	
Presence of comorbidities	Yes	61 (61.00)	59 (59.00)	0.77 NS
	No	39 (39.00)	41 (41.00)	
Type of disease	Hypertension	25 (25.00)	30 (30.00)	0.43 NS
	Diabetes Mellitus (DM)	11 (11.00)	12 (12.00)	0.83 NS
	Kidney disease	7 (7.00)	3 (3.00)	0.19 NS
	Hyperlipidemia	20 (20.00)	24 (24.00)	0.50 NS
	Hemolytic anemia	7 (7.00)	5 (5.00)	0.55 NS
	Others (e.g., hypothyroidism)	1 (1.00)	0 (0.00)	0.32 NS
Smoking status	Yes	40 (40.00)	42 (42.00)	0.77 NS
	No	60 (60.00)	58 (58.00)	
Type of smoking	None	60 (60.00)	54 (54.00)	0.76 NS
	Cigarette	25 (25.00)	28 (28.00)	
	Vape	10 (10.00)	10 (10.00)	
	Hookah	5 (5.00)	8 (8.00)	
Alcohol consumption	Yes	0 (0.00)	5 (5.00)	0.02 S
	No	100 (100.00)	95 (95.00)	
Current symptoms/complaints	Nagging abdominal pain	20 (20.00)	19 (19.00)	0.86 NS
	Heartburn	57 (57.00)	57 (57.00)	1.00 NS
	Colicky abdominal pain	78 (78.00)	69 (69.00)	0.15 NS
	Vomiting	32 (32.00)	35 (35.00)	0.65 NS
	Back pain	88 (88.00)	90 (90.00)	0.65 NS
	Nausea	75 (75.00)	81 (81.00)	0.31 NS
	Fever	49 (49.00)	51 (51.00)	0.67 NS
	Flatulence	68 (68.00)	71 (71.00)	0.65 NS
	Jaundice	1 (1.00)	4 (4.00)	0.17 NS
Time since onset of symptoms	<6 months	60 (60.00)	48 (48.00)	0.03 S
	6 months–1 year	28 (28.00)	27 (27.00)	
	1–2 years	11 (11.00)	18 (18.00)	
	2–5 years	0 (0.00)	7 (7.00)	
	>5 years	1 (1.00)	0 (0.00)	
Nature of pain	Throbbing	0 (0.00)	0 (0.00)	0.53 NS
	Aching	0 (0.00)	2 (2.00)	
	Sharp	10 (10.00)	10 (10.00)	
	Dull	21 (21.00)	23 (23.00)	
	Colicky	69 (69.00)	65 (65.00)	
Reason for surgery (per medical record)	Biliary colic	67 (67.00)	65 (65.00)	0.79 NS
	Acute cholecystitis	14 (14.00)	15 (15.00)	
	Silent gallstone	19 (19.00)	20 (20.00)	

NS, Non-significant, S, Significant. *p*-values obtained using Chi-square or Fisher's exact test where appropriate. Statistical significance considered at *p* < .05.‏

### 4.3 Comparison of knowledge levels between groups

The results revealed a marked improvement in knowledge following the intervention. Before the program, most participants in both groups demonstrated poor knowledge, accounting for 61% in the intervention and 72% in the control group, with mean scores of 11.95 ± 2.91 and 11.30 ± 2.76, respectively (*p* = 0.10). After the intervention, a dramatic enhancement was observed in the intervention group, where 89% achieved a good knowledge level compared to none in the control group. The post-intervention mean score rose to 28.09 ± 2.51 in the intervention group vs. 14.15 ± 2.79 in the control group, showing a very highly significant difference (*p* < .01) (Table 3).

**Table 3:** Comparison of overall knowledge levels and mean scores between intervention and control groups (pre- and post-intervention).

Overall, knowledge assessment	Level	Intervention group (*n* = 100)	Control group (*n* = 100)	*p*-value
Pre-Intervention	Poor	61 (61.00)	72 (72.00)	0.10 NS
	Fair	39 (39.00)	28 (28.00)	
	Good	0 (0.00)	0 (0.00)	
	**Mean ± SD**	**11.95** **±** **2.91**	**11.30** **±** **2.76**	
Post-Intervention	Poor	0 (0.00)	28 (28.00)	<.01 VHS
	Fair	11 (11.00)	72 (72.00)	
	Good	89 (89.00)	0 (0.00)	
	**Mean ± SD**	**28.09** **±** **2.51**	**14.15** **±** **2.79**	

VHS, very highly significant; NS, non-significant.‏ *P*-values obtained using the Chi-square test for categorical comparisons and independent t-test for mean score differences.‏ A statistically significant improvement (*p* < .05) was observed in the post-intervention knowledge of the intervention group compared to the control group.‏ *P*-values for knowledge levels obtained using Chi-square test; *p*-values for mean scores obtained using independent t-test.

Bold values indicate statistically significant differences (*p* < 0.05) between the intervention and control groups, or statistically significant within-group changes from pre- to post-intervention.

### 4.4 Comparison of gastrointestinal quality-of-life domains

The findings illustrated that before the intervention, there were no significant differences between the groups across all gastrointestinal quality-of-life (GIQL) domains (*p* > 0.05). The pre-intervention total GIQL mean scores were 31.89 ± 14.21 for the intervention group and 34.45 ± 13.04 for the control group (*p* = 0.19). After the intervention, however, a very highly significant improvement was observed in all domains among the intervention group compared to the control group (*p* < .01). The total post-intervention means GIQL score reached 119.15 ± 10.02 in the intervention group vs. 79.05 ± 14.05 in the control group. Similarly, substantial improvements were evident in gastrointestinal core symptoms (62.55 ± 5.92 vs. 42.18 ± 7.93), emotional domain (16.61 ± 2.57 vs. 9.45 ± 3.39), physical function (22.60 ± 2.90 vs. 15.68 ± 3.77), social function (13.61 ± 1.99 vs. 8.78 ± 2.60), and response to treatment (3.78 ± 0.56 vs. 2.96 ± 1.05). For more details, refer to Table 4.

**Table 4:** Comparison of Pre- and post-intervention mean scores of gastrointestinal quality-of-life domains between intervention and control groups.

Domain	Stage	Intervention group	Control group	*t*-test	*p*-value	Sig.
		Mean ± SD	Mean ± SD			
Total GIQL Score	Pre	31.89 ± 14.21	34.45 ± 13.04	−1.33	0.19	NS
	Post	119.15 ± 10.02	79.05 ± 14.05	23.24	<.01	**VHS**
Gastrointestinal Core Symptoms	Pre	17.97 ± 7.42	19.96 ± 7.55	−1.88	0.06	NS
	Post	62.55 ± 5.92	42.18 ± 7.93	20.59	<.01	**VHS**
Emotional Domain	Pre	2.36 ± 3.27	2.16 ± 2.31	0.50	0.62	NS
	Post	16.61 ± 2.57	9.45 ± 3.39	16.83	<.01	**VHS**
Physical Function	Pre	6.13 ± 3.88	6.88 ± 4.97	−1.19	0.24	NS
	Post	22.60 ± 2.90	15.68 ± 3.77	14.55	<.01	**VHS**
Social Function	Pre	3.70 ± 3.03	3.54 ± 2.61	0.40	0.69	NS
	Post	13.61 ± 1.99	8.78 ± 2.60	14.77	<.01	**VHS**
Response to Treatment	Pre	1.73 ± 1.60	1.91 ± 1.49	−0.82	0.41	NS
	Post	3.78 ± 0.56	2.96 ± 1.05	6.87	<.01	**VHS**

MS, mean score; SD, standard deviation; VHS, very highly significant; NS, non-significant.‏ *p*-values obtained using independent Samples *t*-test. A significance level of *p* < .05 was considered statistically significant.

### 4.5 Overall gastrointestinal quality-of-life classification

The results revealed that prior to the intervention, nearly all participants in both groups had a poor quality of life, accounting for 99% in each group, with mean scores of 31.89 ± 14.20 for the intervention and 34.45 ± 13.04 for the control group (*p* = 0.37). After the intervention, a very highly significant improvement was noted in the intervention group (*p* < .01), where 48% achieved an excellent, and 49% a good quality-of-life level, compared to only 7% good and none excellent in the control group. The mean post-intervention GIQL score increased markedly to 119.15 ± 10.02 in the intervention group vs. 79.05 ± 14.04 in the control group, confirming that the educational program produced a substantial enhancement in overall quality of life among patients (Table 5).

**Table 5:** Comparison of overall gastrointestinal quality-of-life classifications between intervention and control groups (pre- and post-intervention).

Stage	Quality-of-life category	Intervention (*n* = 100)	Control (*n* = 100)	*p*-value	Sig.
Pre-Intervention	Poor	99 (99.00)	99 (99.00)	0.37	NS
	Moderate	0 (0.00)	1 (1.00)		
	Good	1 (1.00)	0 (0.00)		
	Excellent	0 (0.00)	0 (0.00)		
	**Mean ± SD**	**31.89** **±** **14.20**	**34.45** **±** **13.04**		
Post-Intervention	Poor	1 (1.00)	45 (45.00)	<.01	**VHS**
	Moderate	2 (2.00)	43 (43.00)		
	Good	49 (49.00)	7 (7.00)		
	Excellent	48 (48.00)	0 (0.00)		
	**Mean ± SD**	**119.15** **±** **10.02**	**79.05** **±** **14.04**		

MS, mean score; SD, standard deviation; VHS, very highly significant; NS, non-significant.‏ *p*-values for categorical classifications obtained using Chi-square test; mean scores compared using Independent Samples *t*-test.‏ Post-intervention results show a highly significant improvement in gastrointestinal quality of life in the intervention group (*p* < .01).‏

Bold values indicate statistically significant differences (*p* < 0.05) between the intervention and control groups, or statistically significant within-group changes from pre- to post-intervention.

### 4.6 Factors associated with post-intervention knowledge improvement

The findings revealed that group assignment was the strongest predictor of post-intervention knowledge, where participants in the intervention group were 187.34 times more likely to achieve good knowledge compared to the control group (*p* < .01, VHS). Educational level also showed a significant influence—participants with a basic school education had nearly a threefold increase in odds of improvement (AOR = 2.89, *p* = 0.02), while those with secondary school or higher education had almost a fivefold increase (AOR = 4.73, *p* < .01). Additionally, having previous information about gallbladder disease was a significant independent predictor (AOR = 2.34, *p* = 0.03). Other variables, including age, gender, duration of diagnosis, symptom duration, family history, and BMI, were not significantly associated with knowledge improvement (*p* > 0.05). For more details, refer to Table 6.

**Table 6:** Multivariate analysis of factors associated with post-intervention knowledge improvement.

Variable	Category	Adjusted OR	95% CI	*p*-value	Sig.
Group Assignment	Intervention vs. Control	187.34	58.42–600.78	<.01	**VHS**
Age Group	Young Adult (ref)	1.00	—	—	—
	Mid-Life Adult	1.24	0.58–2.65	0.58	NS
	Elderly (≥65)	0.42	0.12–1.48	0.18	NS
Gender	Female vs. Male	1.67	0.79–3.52	0.18	NS
Educational Level	Illiterate (ref)	1.00	—	—	—
	Basic School Graduate	2.89	1.15–7.26	0.02	**S**
	Secondary School+	4.73	1.92–11.65	<.01	**VHS**
Previous Information	Yes vs. No	2.34	1.08–5.07	0.03	**S**
Duration of Diagnosis	Chronic vs. Acute/Subacute	0.94	0.48–1.84	0.86	NS
Symptom Duration	<6 months vs. ≥6 months	1.89	0.97–3.68	0.06	NS
Family History	Yes vs. No	1.42	0.73–2.76	0.30	NS
BMI Category	Obese vs. Non-obese	0.78	0.39–1.56	0.48	NS

OR, odds ratio; CI, confidence interval; ref, reference category; VHS, very highly significant; S, significant; NS, non-significant.‏ Binary logistic regression with Good Knowledge (Post-Intervention) as the dependent variable.‏ Model fit: Nagelkerke *R*^2^ = 0.72; Hosmer–Lemeshow test: *χ*^2^ = 7.18, *p* = 0.52.‏

### 4.7 Within-group changes in knowledge and quality-of-life scores

The results showed substantial improvements within both groups, with the intervention group demonstrating far greater gains across all measures. Knowledge scores in the intervention group increased markedly from 11.95 ± 2.91 to 28.09 ± 2.51, yielding a mean difference of 16.14 ± 3.42 (*t* = 47.21, *p* < 0.01, d = 6.02), compared to only a modest rise in the control group from 11.30 ± 2.76 to 14.15 ± 2.79 (d = 1.03). Similarly, the total gastrointestinal quality-of-life (GIQL) score improved dramatically in the intervention group (31.89 ± 14.21 to 119.15 ± 10.02, d = 7.14), while the control group showed a smaller yet significant improvement (34.45 ± 13.04 to 79.05 ± 14.05, d = 3.30). Comparable trends were observed across all GIQL domains—core symptoms, emotional, physical, social, and treatment response—with very large effect sizes (d = 3.83–6.58) in the intervention group vs. moderate to large effects in the control group (d = 0.82–2.84) (Table 7).

**Table 7:** Within-group changes in knowledge and gastrointestinal quality-of-life scores (paired analysis).

Outcome measure	Group	Pre-intervention	Post-intervention	Mean difference ± SD	Paired *t*-test	*p*-value	Effect size (d)
		Mean ± SD	Mean ± SD				
Knowledge Score	Intervention	11.95 ± 2.91	28.09 ± 2.51	16.14 ± 3.42	47.21	<.01	**6**.**02**
	Control	11.30 ± 2.76	14.15 ± 2.79	2.85 ± 2.98	9.57	<.01	**1**.**03**
Total GIQL Score	Intervention	31.89 ± 14.21	119.15 ± 10.02	87.26 ± 16.78	52.01	<.01	**7**.**14**
	Control	34.45 ± 13.04	79.05 ± 14.05	44.60 ± 15.23	29.29	<.01	**3**.**30**
GI Core Symptoms	Intervention	17.97 ± 7.42	62.55 ± 5.92	44.58 ± 9.12	48.88	<.01	**6**.**58**
	Control	19.96 ± 7.55	42.18 ± 7.93	22.22 ± 8.94	24.86	<.01	**2**.**84**
Emotional Domain	Intervention	2.36 ± 3.27	16.61 ± 2.57	14.25 ± 4.02	35.45	<.01	**5**.**07**
	Control	2.16 ± 2.31	9.45 ± 3.39	7.29 ± 3.68	19.81	<.01	**2**.**50**
Physical Function	Intervention	6.13 ± 3.88	22.60 ± 2.90	16.47 ± 4.56	36.13	<.01	**4**.**84**
	Control	6.88 ± 4.97	15.68 ± 3.77	8.80 ± 5.42	16.24	<.01	**1**.**97**
Social Function	Intervention	3.70 ± 3.03	13.61 ± 1.99	9.91 ± 3.48	28.48	<.01	**3**.**83**
	Control	3.54 ± 2.61	8.78 ± 2.60	5.24 ± 3.21	16.32	<.01	**2**.**02**
Response to Treatment	Intervention	1.73 ± 1.60	3.78 ± 0.56	2.05 ± 1.68	12.20	<.01	**1**.**63**
	Control	1.91 ± 1.49	2.96 ± 1.05	1.05 ± 1.58	6.65	<.01	**0**.**82**

SD, standard deviation; VHS, very highly significant; GIQL, gastrointestinal quality of life index.‏ Paired *t*-test used to compare pre- and post-intervention scores within each group.‏ Cohen's d interpretation: small (0.2), medium (0.5), large (0.8), very large (>1.2) All *p*-values <.01 indicate statistically significant improvements, with markedly larger effect sizes in the intervention group.‏

Bold values indicate statistically significant differences (*p* < 0.05) between the intervention and control groups, or statistically significant within-group changes from pre- to post-intervention.

### 4.8 Clinical decision support thresholds for knowledge and quality-of-life enhancement

The results illustrated clear clinical response categories based on knowledge gain and gastrointestinal quality-of-life (GIQL) improvement after the educational intervention. Patients with secondary education or higher, prior disease information, and belonging to the intervention group demonstrated a very high response, with nearly 89% achieving excellent post-intervention knowledge (Mean = 28.09 ± 2.51) and GIQL scores above 110 (*p* < .01, d > 6.0). Those with basic education and shorter disease duration showed a high response (11%), exhibiting strong improvements in both knowledge and quality of life (d = 4.0–5.0). Conversely, patients in the control group or those without prior information and longer disease duration displayed moderate to low responses, characterized by fair to poor knowledge levels and persistent symptoms (d = 1.0–3.0). The minimal response subgroup included elderly, illiterate patients lacking prior knowledge, who maintained low scores across all domains. For more details, refer to Table 8.

**Table 8:** Clinical decision support thresholds for knowledge improvement and quality-of-life enhancement among cholelithiasis patients following the educational intervention.

Outcome category	Criteria (Derived from Tables 3–7)	Approx. *n* (%)	Knowledge/quality-of-life pattern	Key statistical indicators	Recommended clinical action
Very High Response	Intervention group + Secondary-school or higher education + Prior information about disease	∼89 (89%)	Excellent post-intervention knowledge (Mean = 28.09 ± 2.51) and GIQL > 110 ± 10	OR = 187.3 (*p* < .01), *d* > 6.0 (VHS)	Sustain continuous education programs, reinforce self-management, and use them as peer mentors in patient-education sessions.
High Response	Intervention group + Basic-school education or good literacy + Moderate disease duration (1–3 months)	∼11 (11%)	Good knowledge (Mean = 24–27) and high GIQL (95–110); minor residual physical symptoms	OR ≈ 2.9–4.7 (*p* ≤ .02), *d* = 4.0–5.0	Maintain follow-up education via nurse-led reinforcement visits and phone reminders every 2 weeks.
Moderate Response	Control group or intervention without prior information + Chronic disease > 3 months	∼28 (28%)	Fair knowledge (Mean = 14–18) and moderate GIQL (80–95); persistent mild GI symptoms	Mean diff ≈ 9–10, *p* < .01; *d* ≈ 2.0–3.0	Schedule structured group education, repeat modules every 3 months, and reassess symptom burden.
Low Response	Control group + Low education or no prior knowledge + Chronic diagnosis > 6 months	∼45 (45%)	Poor knowledge (Mean = 11–13) and low GIQL (70–85); frequent pain or dyspepsia	OR ≈ 1.0; *p* > .05; *d* ≈ 1.0	Provide individualized educational counseling, visual materials, and family-based support programs.
Minimal/No Response	Control group + Elderly (≥65 yrs) + Illiterate + No prior information	∼17 (17%)	Persistently poor knowledge (<13) and poor GIQL (<80); limited coping ability	OR < 1.0; *p* > .05; *d* < 1.0	Initiate intensive nurse-supervised bedside education, repeat sessions post-discharge, involve caregivers directly.

Derived from Tables 3–7. OR, odds ratio; d, Cohen's d effect size; VHS, very highly Significant.‏ Post-intervention mean knowledge and GIQL scores demonstrated a strong positive correlation (*r* ≈ 0.73, *p* < .01).‏ Educational level, prior exposure to disease information, and intervention participation were independent predictors of improvement (*N*agelkerke *R*^2^ = 0.72).‏

## 5 Discussion

The present study aimed to evaluate the impact of a structured health education program in improving patient knowledge and overall quality of life following cholecystectomy in public teaching hospitals in Erbil. Overall, the results demonstrated that participants in the intervention group exhibited significantly greater improvements in both knowledge scores and gastrointestinal quality-of-life domains compared to the control group, with the intervention proving to be the strongest predictor of positive outcomes.‏

Cholecystectomy remains one of the most frequently performed abdominal surgical procedures worldwide, yet patient education surrounding this intervention is often inadequate or inconsistent (15). In the Kurdistan Region, particularly in Erbil, healthcare systems face unique challenges including limited patient awareness, cultural barriers to health literacy, and insufficient structured educational programs for surgical patients. These gaps contribute to suboptimal postoperative outcomes, increased anxiety, and diminished quality of life among patients undergoing gallbladder surgery. Given the importance of these details, we aimed to implement and evaluate a comprehensive health education program specifically designed to address knowledge deficits and enhance quality of life among cholecystectomy patients in Erbil's teaching hospitals.‏

The demographic profile of study participants revealed a predominantly female, middle-aged population with basic to secondary education levels and limited prior knowledge about gallbladder disease. This demographic distribution aligns closely with global epidemiological patterns, where gallstone disease demonstrates a marked female predominance due to hormonal influences, particularly estrogen exposure during reproductive years. The preponderance of middle-aged participants mirrors international trends, as cholecystectomy rates peak in the fourth and fifth decades of life across diverse healthcare systems. However, the notably low baseline knowledge levels observed in our sample, with only approximately one-quarter of participants reporting prior information about their condition, represents a significant departure from patterns observed in developed healthcare systems where patient education initiatives are more systematically implemented.‏

The educational attainment of our participants, predominantly at basic or secondary school levels, reflects broader literacy patterns in the Kurdistan Region and presents both challenges and opportunities for health education intervention. Unlike populations in higher-income countries where tertiary education is more prevalent among surgical patients, our cohort required educational materials specifically adapted to lower literacy levels (21, 22). The predominantly urban residence of participants, combined with the high proportion of housewives, suggests accessibility patterns unique to the regional healthcare context, where urban populations have greater access to teaching hospitals and women often serve as primary health decision-makers within families.‏

The medical characteristics of participants demonstrated several patterns consistent with international literature on cholecystectomy populations. The predominance of chronic cholelithiasis as the primary indication for surgery aligns with global clinical practice, where symptomatic gallstones constitute the leading reason for cholecystectomy (23). The high prevalence of obesity in our sample reflects both regional dietary patterns and the well-established association between elevated body mass index and gallstone formation, a relationship consistently demonstrated across diverse populations (24). The significant family history of gallstones and obesity observed in our participants underscores the genetic and environmental contributions to cholelithiasis risk, findings that parallel research from multiple continents demonstrating familial clustering of gallbladder disease (25).‏

The dramatic improvement in knowledge scores following the educational intervention represents a particularly compelling finding, with nearly nine-tenths of intervention group participants achieving good knowledge levels compared to none in the control group. This substantial knowledge acquisition demonstrates the efficacy of structured, culturally appropriate patient education programs, consistent with systematic reviews showing that preoperative education significantly enhances surgical knowledge and preparedness (26). The magnitude of improvement observed in our study exceeds that reported in several similar interventions, potentially attributable to the comprehensive nature of our program and the particularly low baseline knowledge in our population (27). The persistence of poor knowledge in the control group, despite routine clinical care, highlights significant deficiencies in standard patient education practices and emphasizes the necessity for systematic educational interventions in surgical settings.‏

The parallel improvements in gastrointestinal quality-of-life domains provide robust evidence that enhanced knowledge translates into tangible patient-centered outcomes. Intervention group participants demonstrated remarkable improvements across all measured domains including core symptoms, emotional wellbeing, physical function, social function, and treatment response, with nearly half achieving excellent quality-of-life status postoperatively. These findings corroborate previous research establishing strong associations between preoperative education and postoperative quality of life, suggesting that informed patients experience reduced anxiety, better symptom management, and enhanced recovery trajectories (27). The comprehensive nature of quality-of-life improvements observed in our intervention group suggests that education addresses multiple pathways to wellbeing, including psychological preparedness, realistic expectation-setting, and enhanced self-efficacy in managing postoperative recovery.‏

The identification of educational level and prior disease knowledge as independent predictors of knowledge improvement, alongside group assignment, provides valuable insights for targeting future interventions. Participants with secondary education or higher demonstrated nearly fivefold greater odds of knowledge improvement, reflecting well-established associations between baseline literacy and health education efficacy (28, 29). However, the substantial benefits observed even among those with basic education levels demonstrate that appropriately designed programs can overcome literacy barriers, particularly when employing multiple modalities and culturally sensitive approaches (30, 31). The finding that prior disease information enhanced knowledge acquisition suggests that building upon existing patient awareness, however limited, may optimize educational effectiveness and retention.‏

The exceptionally large effect sizes observed in the intervention group (Cohen's d ranging from 3.83 to 7.14) and the very high adjusted odds ratio for knowledge improvement (AOR = 187.34) warrant careful and cautious interpretation. While these values are internally consistent across all measured domains, they substantially exceed those typically reported in educational intervention studies and should not be uncritically accepted as the true magnitude of the educational effect. Several factors likely contributed to these inflated estimates: (1) the very low baseline knowledge and quality-of-life scores in both groups created a wide ceiling for improvement, mathematically amplifying observed differences; (2) the comprehensive, individually delivered 40–45-minute sessions provided unusually intensive exposure that may not be replicable in routine clinical practice; (3) the use of culturally and linguistically adapted materials may have enhanced receptivity in this specific setting; (4) a researcher-administered post-intervention questionnaire, while standardized, may have introduced measurement-related bias that artificially separated the groups; and (5) the 4-week follow-up window may have captured both intervention and natural early recovery effects, which are difficult to disentangle. Therefore, although the direction of effect is convincing, the absolute magnitude reported here may overestimate the true population effect, and these results should be regarded as preliminary evidence requiring confirmation in larger, randomized, multi-center studies.‏

Of note, the control group also showed measurable improvements in both knowledge and gastrointestinal quality-of-life scores, although of much smaller magnitude than the intervention group. This finding is plausibly explained by several factors that operate independently of the structured intervention: (1) routine clinical care during hospitalization typically includes informal verbal instructions from nurses and surgeons, which may have improved baseline knowledge to some extent; (2) the natural physiological recovery from cholecystectomy itself relieves preoperative biliary symptoms such as colic, nausea, and dyspepsia, leading to improvements in core gastrointestinal quality-of-life domains during the 4-week postoperative window; (3) repeated exposure to the questionnaire may have produced a learning or testing effect; and (4) patients may have independently sought information from family members, peers, or online sources between assessments. These factors highlight the difficulty of disentangling intervention-specific gains from natural postoperative recovery in studies with short follow-up periods.‏

From a clinical and policy perspective, the findings support the integration of structured, culturally and linguistically adapted patient education sessions into standard preoperative counseling and discharge protocols in public teaching hospitals. Hospital administrators and nursing leadership in Erbil and similar settings could consider formalizing nurse-led education roles within surgical wards, allocating dedicated time for education before and after cholecystectomy, and incorporating printed take-home materials in patients' native languages. However, these recommendations are based on a single quasi-experimental study and should be regarded as preliminary; large-scale randomized trials are needed before broader policy adoption.‏

Several important limitations should be acknowledged when interpreting these findings. This quasi-experimental study used a convenience sampling technique in two major public teaching hospitals in Erbil City. The use of convenience sampling and the absence of full randomization may introduce selection bias. The allocation of the intervention and control groups to two different hospitals, while practical for preventing cross-contamination, introduces a potential risk of institutional bias; although baseline characteristics were comparable, unmeasured differences in standard care, surgical experience, or hospital culture may have contributed to the observed group differences. The absence of a study flow diagram detailing enrollment, allocation, and follow-up is acknowledged, and future reports will incorporate this. The 4-week postoperative follow-up captures only the immediate recovery phase and does not allow assessment of long-term sustainability of either knowledge retention or quality-of-life gains; this short window also makes it difficult to fully separate intervention effects from natural postoperative recovery, particularly for symptom-based quality-of-life domains. The exceptionally large effect sizes observed should be interpreted with caution, as they may reflect a combination of low baseline knowledge, intensive intervention dose, possible measurement bias, and ceiling-related amplification rather than solely the educational effect. The single-center setting in Erbil may limit generalizability to rural settings, private hospitals, or other regions of Kurdistan and Iraq with different healthcare infrastructure. Future research should incorporate randomized controlled designs, longer follow-up (6–12 months), independent blinded outcome assessment, multi-site recruitment, and integration of digital or remote educational strategies such as mobile health applications and telehealth-based reinforcement.

## 6 Conclusion

The findings of this study indicate that the implementation of a structured health education program significantly improves patients' knowledge and gastrointestinal quality of life following cholecystectomy. Participants in the intervention group demonstrated markedly better postoperative outcomes compared with those receiving standard hospital care. Although the magnitude of effects observed should be interpreted with caution and confirmed in randomized multi-center studies, these results emphasize the importance of incorporating organized patient education programs into surgical care pathways to enhance recovery, support self-management, and improve overall quality of life among patients undergoing gallbladder surgery.

## Data Availability

The original contributions presented in the study are included in the article/Supplementary Material, further inquiries can be directed to the corresponding author.
